# CO_2_-Driven N-Formylation/N-Methylation of Amines Using C-Scorpionate Metal Complexes

**DOI:** 10.3390/molecules29040870

**Published:** 2024-02-16

**Authors:** Inês A. S. Matias, Anna M. Trzeciak, Paulina Pąchalska, Ana P. C. Ribeiro, Luísa M. D. R. S. Martins

**Affiliations:** 1 Centro de Química Estrutural, Institute of Molecular Sciences, Departamento de Engenahria Química, Instituto Superior Técnico, Universidade de Lisboa, 1049-001 Lisbon, Portugal; ines.matias@tecnico.ulisboa.pt (I.A.S.M.); apribeiro@ist.utl.pt (A.P.C.R.); 2 Faculty of Chemistry, University of Wrocław, 14 F. Joliot-Curie, 50-383 Wrocław, Poland; paulina.pachalska@uwr.edu.pl

**Keywords:** CO_2_, C-scorpionate, hydrotris(1H-pyrazol-1-yl)methane, catalyst, amine, N-formylation, N-methylation

## Abstract

C-scorpionate metal complexes, specifically, [NiCl_2_(tpm)]·3H_2_O, [CoCl_2_(tpm)]·3H_2_O and [PdCl_2_(tpm)] [tpm = hydrotris(1H-pyrazol-1-yl)methane], were effective in the N-formylation and N-methylation of amines using carbon dioxide, as carbon source, in the presence of sodium borohydride. Various parameters were studied, including reaction time, temperature, solvent volume, presence of additives, and catalyst amount. These parameters were found to have a significant impact on the selectivity of the product. [NiCl_2_(tpm)]·3H_2_O exhibited good conversion at 80 °C, but its selectivity towards formamide decreased with prolonged reaction time. Increasing the amount of [NiCl_2_(tpm)]·3H_2_O, the selectivity changed. [PdCl_2_(tpm)] showed different selectivity compared to [NiCl_2_(tpm)]·3H_2_O, while [CoCl_2_(tpm)]·3H_2_O presented poor results. Monitoring the reaction course by ^1^H NMR revealed the presence of an intermediate species that influenced product formation. These results highlight the versatility and catalytic potential of C-scorpionate metal complexes in the N-formylation/N-methylation of amines in the catalytic system (NaBH_4_/MeCN/CO_2_).

## 1. Introduction

Nowadays, society is confronted with one of the most challenging problems of all, the mitigation of greenhouse gases. The main cause of global warming and ocean acidification is anthropogenic emissions of carbon dioxide released from industries. Consequently, research on carbon dioxide fixation has become one of the most appealing topics for both academia and industry [1]. In fact, CO_2_-based chemicals can reduce the impacts of global warming and fossil depletion through the replacement of traditional petrochemical feedstock processes.

**Scheme 1:**
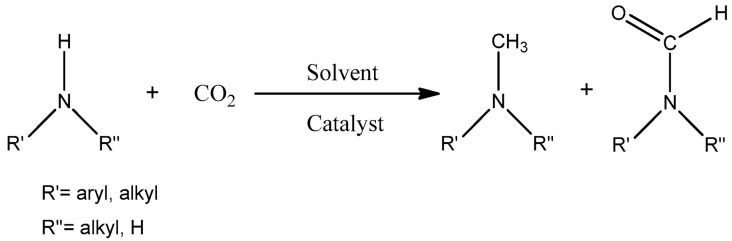
General reaction of N-formylation and N-methylation of amines with CO_2_.

In this work, our focus was on the functionalization of N-methylaniline using carbon dioxide as a greener and more sustainable carbon source to obtain N,N-dimethylaniline and N-methyl-N-phenylformamide (see the general reaction in Scheme 1). These compounds have numerous applications, including dyes, drug components, fragrances, and feedstock for agrochemical production, among others [2,3,4]. Thus, the proposed route, if successful, would consist of an important improvement on the sustainability of the fabrication processes of the above industrial products, while contributing to mitigate the CO_2_ greenhouse effects.

However, the functionalization of N-methylaniline process can be demanding due to the high stability of carbon dioxide as a molecule. To form the characteristic C-N bond found in the desired products, a reducing agent such as hydrosilane, borane, or H_2_ is required [5,6,7].

The selectivity of the reaction has also been a significant concern; for example, in 2020, Zhao et al. presented a selective N-methylation of N-methylaniline with carbon dioxide and H_2_ over a TiO_2_-supported PdZn catalyst. The authors achieved a selectivity of 98.4 for N,N-dimethylaniline, however, with only 15.6% conversion of N-methylaniline, using drastic conditions: 1.5 MPa of CO_2_, 4.5 MPa of H_2_, *n*-octane as solvent at 180 °C [8].

In 2013, Beller et al. reported a selective ruthenium-catalyzed methylation of amines with carbon dioxide and hydrogen, in which a very good yield of 96% of N,N-dimethylaniline and 4% of N-methylaniline were obtained. However, the reaction conditions used in this study (20 atm carbon dioxide, 60 atm of hydrogen, 1.5 mol% of methanesulfonic acid in THF at 140 °C) are too aggressive [9]. Thus, the development of new catalytic systems is important to achieve better selectivity under milder reaction conditions.

Various catalytic systems have been tested for the functionalization of N-methylaniline reaction, including organocatalysts involving superbases (such as N-heterocyclic carbenes, B(C_6_F_5_)_3_, or ionic liquids) [10,11,12,13], as well as metal catalysts such as Ru, Pd, Cu, Rh, and others [14,15,16,17]. Comparing the different catalytic systems, organocatalysts have shown moderate activity, while metal complexes have demonstrated remarkable activity. However, it is important to note that most of these catalytic systems [10,11,12,13,14,15,16,17] require high temperature and pressure (using hydrogen as the reductant) and have low atom economy (using hydrosilanes as reductants).

Additionally, metal-free or catalyst-free examples of N-formylation and N-methylation of amines with carbon dioxide have been achieved by using boron compounds as reducing agents [18,19,20,21]. Unlike hydrosilanes, metal borohydrides are less expensive reductants that can react with carbon dioxide to form formate borohydride species. However, as reported by You-Ting Wu et al. in 2017, who developed a catalyst free *N*-formylation of amines using CO_2_ and NH_3_BH_3_, the reaction does not selectively yield methylated derivatives [22].

**Figure 1:**
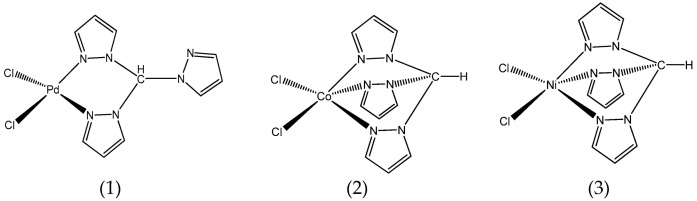
C-scorpionate metal complexes synthesized and used in this work: (1) [PdCl_2_(tpm)]; (2) [CoCl_2_(tpm)]·3H_2_O; (3) [NiCl_2_(tpm)]·3H_2_O, [tpm = hydrotris(1H-pyrazol-1-yl)methane].

Inspired by this, we have decided to explore the catalytic activity of three different metal complexes bearing the simplest C-scorpionate ligand (tpm, Figure 1), [NiCl_2_(tpm)]·3H_2_O, [CoCl_2_(tpm)]·3H_2_O and [PdCl_2_(tpm)] [tpm = hydrotris(1H-pyrazol-1-yl)methane], in the N-formylation and N-methylation of amines with carbon dioxide, using sodium borohydride (NaBH_4_) as a reducing agent, under mild reaction conditions, and employing a less toxic solvent such as acetonitrile instead of 1,4-dioxane [23].

C-scorpionates are poly(1H-pyrazol-1-yl)methane pro-ligands that consist of two or more N-deprotonated pyrazole rings (pyrazolyl, pz) bounded to a carbon atom by one of the nitrogen atoms presented in the ring. The designation “scorpionate” is due to the ligand structure and binding mode to a metal center to form a coordination compound; that is, the nitrogen heteroatoms of the pyrazolyl rings are able to occupy two (κ^2^-coordination mode) or three (κ^3^-coordination mode) facially adjacent vacant positions of the coordination sphere of a metal center.

C-homoscorpionate (bearing three pyrazolyl moieties) tris(1H-pyrazol-1-yl)methane [RC(R’pz)_3_ (pz = pyrazolyl; R = H or substituent at the methine carbon; R’ = H or substituent at the pz ring], metal complexes were successfully applied as catalysts in different types of reactions, for example, (i) oxidations[24] (alkane, alkene, alcohol or ketone), (ii) hydrocarboxylations of alkanes to carboxylic acids [25], and (iii) catalytic hydrogenation of carbon dioxide into methanol [26], but they have never been tested in the N-formylation and N-methylation of amines with carbon dioxide.

It is also important to mention that, to our knowledge, no examples of this catalytic system (NaBH_4_/MeCN/CO_2_) using homogeneous metal complexes have been found in the literature. Thus, this work addresses the contribution of transition metal complexes bearing homoscorpionate tris(1H-pyrazol-1-yl)methane type ligands in the design of catalytic innovative processes for industrially significant reactions aiming at the development of eco-benign and clean synthetic methodologies.

## 2. Results and Discussion

**Scheme 2:**
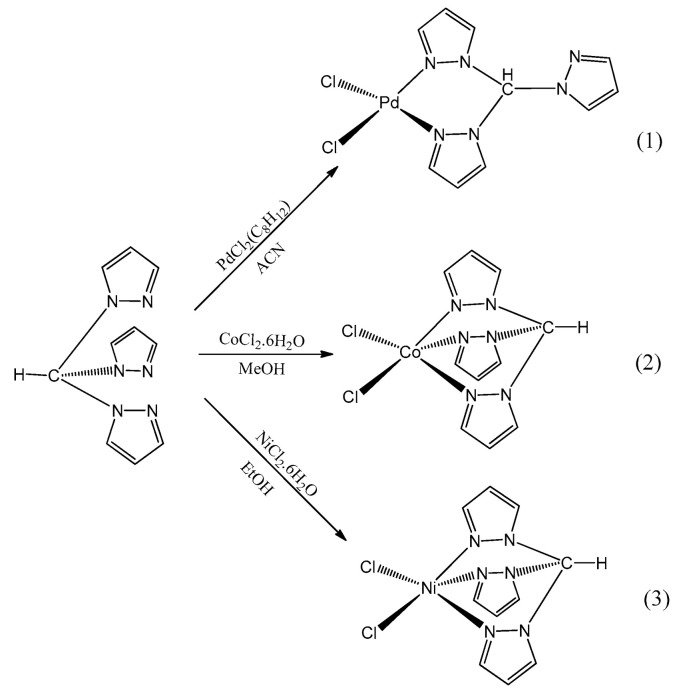
Synthesis of the C-scorpionate metal complexes (**1**)–(**3**) used in this work.

The synthesis of the C-scorpionate metal complexes used in this study is presented in Scheme 2.

The structures of the above C-scorpionate complexes (**1**) to (**3**) were proposed based on their structural characterization by different techniques such as FTIR and elemental analysis. Additionally, in the case of the palladium C-scorpionate complex (**1**), it was also characterized by single crystal X-ray and, once it is a diamagnetic compound, nuclear magnetic resonance spectroscopy.

The palladium C-scorpionate complex (**1**) exhibits a κ^2^-coordination mode, where the nitrogen heteroatoms of the pyrazolyl rings are able to occupy two facially adjacent vacant positions of the coordination sphere of the palladium metal center. However, in the case of the other two C-scorpionate metal complexes, cobalt (**2**) and nickel (**3**), the κ^3^-coordination mode of the pyrazolyl rings to the metal center was the preferred.

**Scheme 3:**
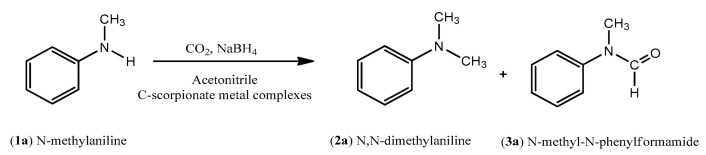
N-formylation and N-methylation of N-methylaniline with CO_2_ and NaBH_4_, catalyzed by C-scorpionate metal complexes.

The catalytic performance of C-scorpionate metal complexes (**1**)–(**3**) was evaluated for the formylation and methylation of N-methylaniline (**1a**) with carbon dioxide and sodium borohydride (Scheme 3).

During our investigation into the conversion of carbon dioxide, we verified that both N,N-dimethylaniline (**2a**) and N-methyl-N-phenylformamide (**3a**) can be simultaneously obtained when NaBH_4_ is used as the reducing agent.

Initial experiments revealed that the use of acetonitrile as solvent yielded better results compared to 1,4-dioxane in terms of substrate conversion and the reductant-to-substrate ratio. This finding prompted us to focus on enhancing the selectivity of the reaction.

Various parameters were explored, as outlined in Table 1 and Table 2, including the reaction time, temperature, solvent volume, presence of additives, and catalyst amount. These parameters were found to have a significant impact on the selectivity of the product.

Interestingly, we observed that N-methylaniline can be efficiently converted to N,N-dimethylaniline and N-methyl-N-phenylformamide under mild reaction conditions, utilizing 5 bar of carbon dioxide and temperatures ranging from 30 to 80 °C.

Different experiments without the use of catalysts or using additives such us triethylamine (Et_3_N) and potassium carbonate (K_2_CO_3_) (entries 1–6, Table 1) were run and, confirming what is reported in the literature, the conversion of N-methylaniline occurs. Contrasting the results obtained by using additives (entries 1 and 2, illustrated in Table 1) with the “blank” experiment (entry 3, Table 1), this indicates that the presence of additives tends to lead to less favorable outcomes, resulting in lower conversions. This observation highlights the potential negative effect of additives in the context of this specific reaction. Furthermore, a direct comparison between the utilization of Et_3_N and K_2_CO_3_ affords a valuable insight into their relative efficacy. This suggests that K_2_CO_3_ is more advantageous in terms of promoting higher conversions compared to Et_3_N.

**Figure 2:**
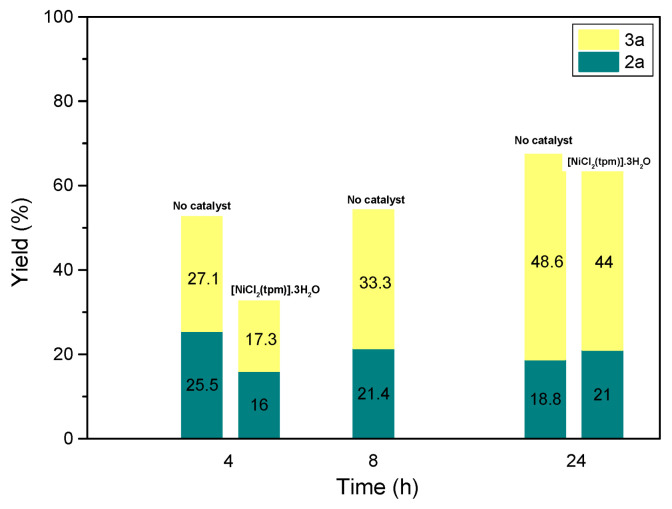
Influence of the reaction time in the conversion of **1a**, with 1 mol% of [NiCl_2_(tpm)]·3H_2_O and without catalyst, using 0.5 mL of solvent at 30 °C.

Additionally, when the reaction time was reduced in the blank experiments (entries 3 and 4, Table 1), both the conversion and the selectivity towards **3a** decreased (Figure 2).

Nevertheless, it is important to acknowledge that the selectivity achieved in the abovementioned experiments was poor, and considering this, we have decided to test the potential of C-scorpionate metal complexes as catalysts, with the objective of enhancing the selectivity of the reaction (entries 7–14, Table 1).

**Figure 3:**
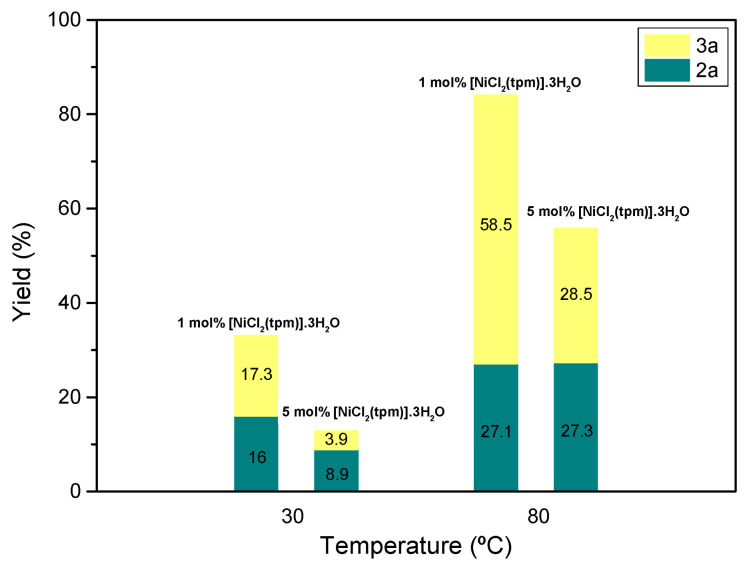
Influence of the temperature in the conversion of **1a**, for different amounts of [NiCl_2_(tpm)]·3H_2_O (1 mol% and 5 mol%), using 0.5 mL of solvent.

In our initial experiments conducted at 30 °C for a 4 h reaction time, using 0.5 mL of acetonitrile, we observed a relatively lower conversion of the substrate. The highest conversion achieved was 33.3% with a ratio of **2a**/**3a** of 0.48/0.52, using 1 mol% of [NiCl_2_(tpm)]·3H_2_O as the catalyst (entry 8, Table 1). Building upon these initial results with [NiCl_2_(tpm)]·3H_2_O, we decided to maintain the same reaction conditions but increase the temperature to 80 °C (Figure 3).

Interestingly, at 80 °C, we observed a significantly improved conversion of 85.6% with a ratio of **2a**/**3a** of 0.32/0.68 (entry 10, Table 1). Encouraged by these findings, we further extended the reaction time from 4 to 24 h, keeping the temperature at 80 °C. The conversion increased even further, reaching 97.6%. However, the selectivity towards formamide decreased, and the ratio of **2a**/**3a** became 0.47/0.53 (entry 13, Table 1).

**Figure 4:**
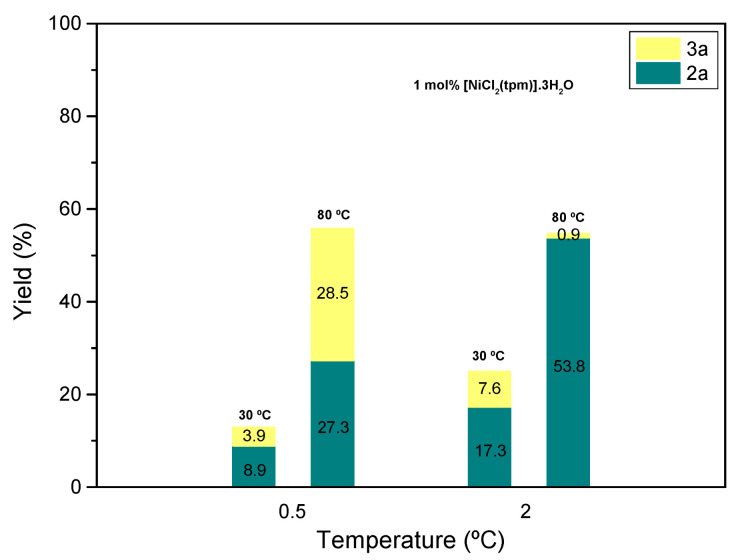
Influence of the volume of solvent in the conversion of **1a**, at different temperatures (30 and 80 °C) for 1 mol% of [NiCl_2_(tpm)]·3H_2_O.

These results indicate that the selectivity of the reaction towards formamide favors higher temperatures for shorter reaction times. Although a longer reaction time leads to increased conversion, it also reduces the selectivity towards N-methyl-N-phenylformamide (compare entries 10 and 13, Table 1). Therefore, optimizing the reaction conditions requires a careful balance between conversion and selectivity. Also, the amount of solvent was investigated (Figure 4), revealing that higher volumes of acetonitrile promote a good conversion of the substrate 81.6% keeping a ratio **2a**/**3a** of 0.29/0.71 (entry 14, Table 1).

Hence, considering these initial studies, we have decided to continue to investigate the selectivity of this reaction by increasing the amount of catalyst from 1 to 5 mol% (Table 2).

It is indeed interesting to note the effects of changing the catalyst loading and reaction conditions on the selectivity of the reaction. When using [NiCl_2_(tpm)]·3H_2_O as the catalyst and optimizing the conditions (4 h, 80 °C, 2 mL of acetonitrile), increasing the catalyst loading from 1 to 5 mol% resulted in a decrease in substrate conversion (entry 3, Table 2). However, there was a significant increase in selectivity towards N,N-dimethylaniline, with a ratio of **2a**/**3a** of 0.98/0.02. Lowering the temperature to 30 °C led to a decrease in substrate conversion, as expected, and a corresponding decrease in the ratio of **2a**/**3a** to 0.69/0.31 (entry 6, Table 2).

**Figure 5:**
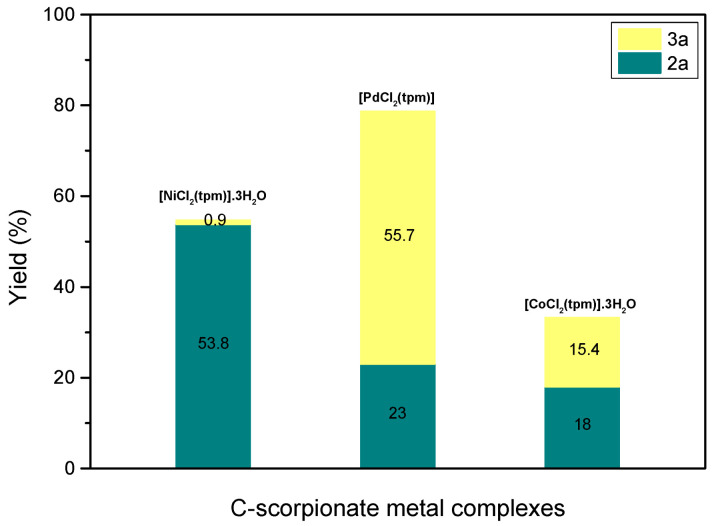
Influence of the different C-scorpionate metal complexes in the conversion of **1a**, for the optimal conditions found (4 h, 80 °C, 2 mL of acetonitrile).

In the case of the palladium scorpionate complex, using 5 mol% of [PdCl_2_(tpm)] under optimal conditions resulted in a conversion of 78.7% with a ratio of **2a**/**3a** of 0.29/0.71, indicating a different selectivity profile compared to the [NiCl_2_(tpm)]·3H_2_O catalyst. However, when [CoCl_2_(tpm)]·3H_2_O was used as the catalyst under the optimal conditions, both the conversion of N-methylaniline and the selectivity of the reaction were poor (Figure 5). Therefore, in terms of achieving better selectivity, the best catalyst is [NiCl_2_(tpm)]·3H_2_O.

To gain further insights into the reaction progress and selectivity, we monitored the reaction course by ^1^H NMR. Various reaction times ranging from 10 min to 24 h were studied under the found optimal conditions (5 mol% [NiCl_2_(tpm)]·3H_2_O, 80 °C, and 0.5 mL CD_3_CN). Based on the obtained spectra, we observed that the product **3a** appeared with higher intensity as early as 10 min reaction, while the peak corresponding to the substrate started to decrease around 30 min of reaction time, indicating the formation of product **2a**.

**Figure 6:**
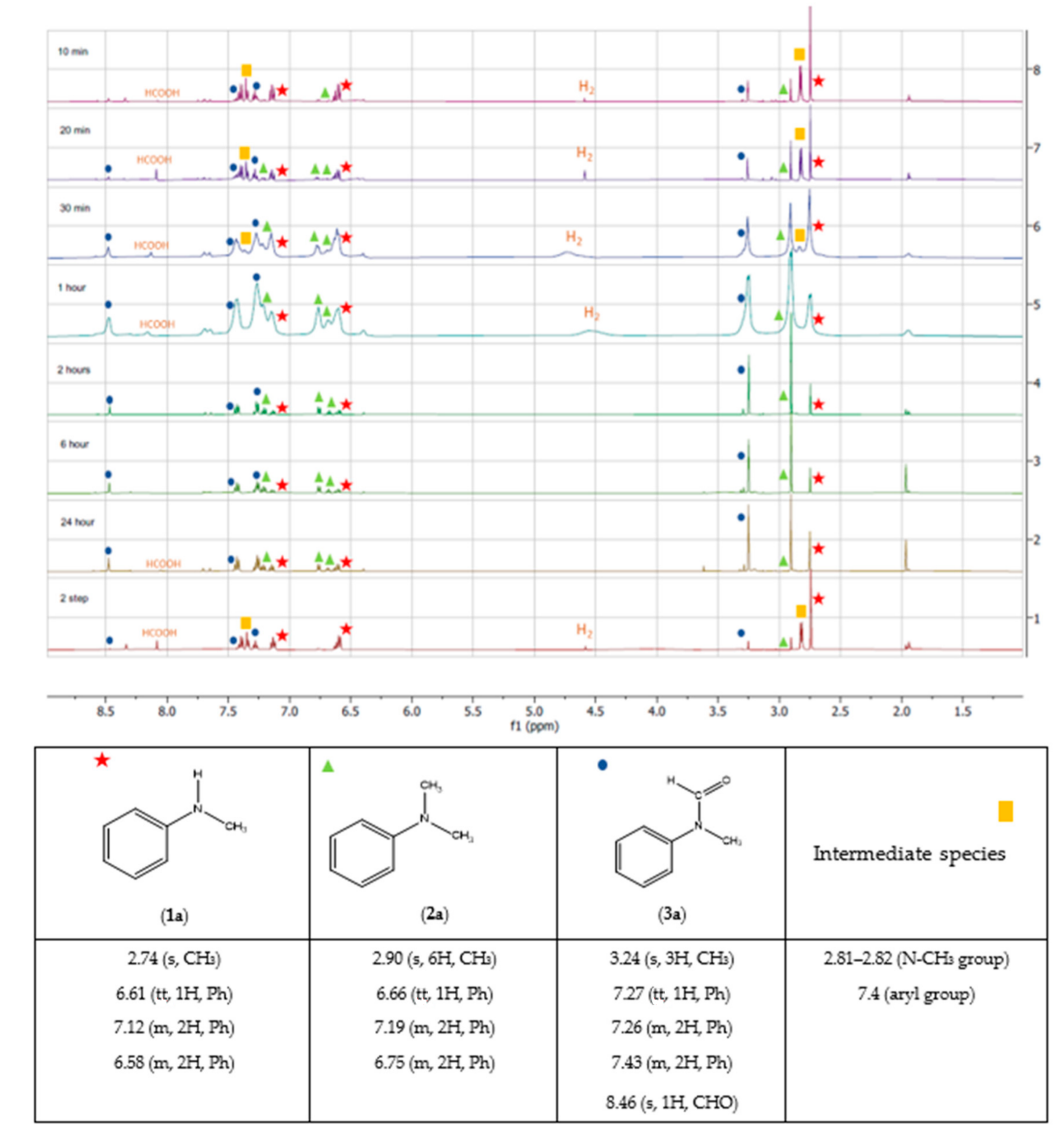
Comparison between all ^1^H NMR spectra and identification of the peaks correspondent to substrate, products, and intermediate species.

These observations from the ^1^H NMR analysis provide valuable information about the reaction kinetics and the order of product formation, shedding light on the selectivity trends observed under different conditions and reaction times (Figure 6).

**Scheme 4:**
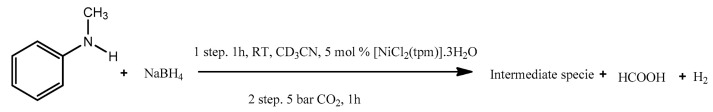
Two-step reaction performed to identify the intermediate species.

Performing a control experiment to investigate the nature of the observed intermediate species was the next approach. Dividing the reaction into two steps (Scheme 4) allowed the identification of the intermediate formed. A similar intermediate was observed in the non-catalyzed methylation/formylation of N-methylaniline and it was identified as an amine adduct of BH_3_ (**1a**-BH_3_) [21].

In the control experiment, the first step involved reacting the substrate with NaBH_4_ in the presence of [NiCl_2_(tpm)]·3H_2_O for 1 h at room temperature. This step likely generates the intermediate species. In the second step, carbon dioxide is added to the reaction mixture, and the reaction is allowed to continue for an additional hour.

This approach allows for a more detailed investigation of the reaction pathway and the role of each reactant in the formation and transformation of the intermediate species.

The observation that the peaks corresponding to the intermediate species disappear between 30 min to 1 h of reaction, coinciding with the appearance of peaks corresponding to product **2a**, suggests that the intermediate species may play a role in promoting the formation of **2a**. This indicates that the intermediate species might be involved in a reaction pathway leading to the desired product.

Furthermore, the presence of a singlet at 4.57 ppm, which corresponds to H_2_, is consistent with the use of sodium borohydride as a reducing agent, which can generate H_2_ during the reaction.

**Scheme 5:**
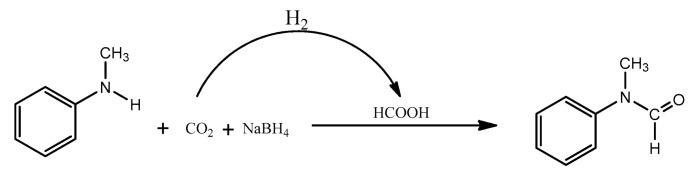
Possible scheme for the synthesis of **3a**.

The peak at 8.05 ppm indicating the presence of formic acid (HCOOH) is interesting and could indicate a side reaction, promoted by the H_2_ formed in situ with the CO_2_, and its presence could contribute to the formation of byproducts, namely **3a** (Scheme 5).

These observations from the NMR spectra provide valuable insights into the reaction mechanism and the involvement of different species during the course of the reaction. Further investigations and characterization of the intermediate species and the role of H_2_ and formic acid could help in understanding the reaction pathways and optimizing the selectivity of the desired product.

A substrate scope was performed, where aniline, piperidine and diphenylamine were tested, using the optimal conditions achieved before. The obtained yields are presented below, in Table 3.

As expected, the lower conversion obtained using diphenylamine as substrate can be explained by the presence of the bulky phenyl groups in the molecule, when compared to the other studied substrates, impairing the interaction with the C-scorpionate catalyst.

## 3. Materials and Methods

### 3.1. Materials

Sodium borohydride, amines, tetra-n-butylammonium bromide, pyrazole, sodium carbonate, chloroform, methanol, diethyl ether, cobalt(II) chloride hexahydrate, and nickel(II) chloride hexahydrate were obtained from Sigma-Aldrich and Merk and used without further purification or drying.

### 3.2. Synthesis of C-Scorpionate Metal Complexes

**Scheme 6:**
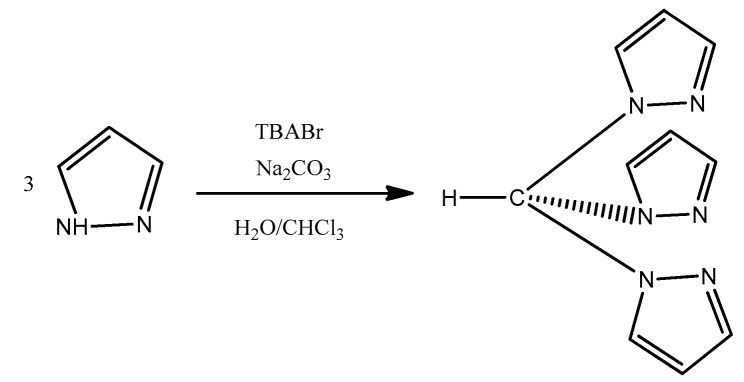
Synthesis of hydrotris(pyrazol-1-yl)methane (tpm).

Hydrotris(1H-pyrazol-1-yl)methane, HC(pz)_3_ (pz = pyrazolyl), was synthesized according to the literature [27]. The synthetic method used was adapted from the one developed by Reger and co-workers (Scheme 6), and consisted by mixing, in a round-bottom flask, pyrazole (73 mmol), tetra-n-butylammonium bromide (3.64 mmol), and distilled water (73.5 mL). The reaction mixture was left under vigorous stirring, and an excess of sodium carbonate was gradually introduced to the flask with continuous stirring to enhance reaction efficiency. After cooling to room temperature, chloroform (36.75 mL) was added. The resulting mixture was left under reflux for 3 days.

After the reaction time was completed, the mixture cooled to room temperature, was filtrated to eliminate the base in excess, and diethyl ether and distilled water were added to the filtrate. After several extractions, the collected organic layer was washed with a saturated brine solution. Activated charcoal was applied to the organic layer, which was then dried over sodium sulfate. After filtration, the solvent was evaporated, and the resulting yellow solid was dried under vacuum.

[CoCl_2_(tpm)]·3H_2_O was synthesized by slowly adding a methanolic solution of hydrotris(1H-pyrazol-1-yl)methane (0.47 mmol) to a methanolic solution of cobalt(II) chloride hexahydrate (0.47 mmol). The mixture was stirred at room temperature for 24 h, resulting in a change in color from pink/red to salmon. The formed precipitate was subsequently collected and rinsed with diethyl ether and the obtained yield was 83%. Elemental analysis required for C_10_H_16_Cl_2_N_6_O_3_Co: C 30.17, H 4.05, N 21.11; found: C 30.82, H 3.60, N 21.53%. FAR-IR (CsI pellet) (cm^−1^): 215 sp and 245 sp [ט (Co-Cl)].

To obtain [NiCl_2_(tpm)]·3H_2_O, a similar procedure was employed. A solution of hydrotris(1H-pyrazol-1-yl)methane (0.47 mmol) in ethanol was dropwise added to an ethanolic solution of nickel(II) chloride hexahydrate (0.47 mmol). The reaction mixture was stirred at room temperature for 24 h, leading to a color transition from green to light blue. The resulting precipitate was collected and washed using diethyl ether. The obtained yield was 70%. Elemental analysis required for C_10_H_16_Cl_2_N_6_O_3_Ni: C 30.19, H 4.05, N 21.12; found: C 30.33, H 3.87, N 21.12%. FAR-IR (CsI pellet) (cm^−1^): 229 sp and 256 sp [ט (Ni-Cl)].

For the synthesis of [PdCl_2_(tpm)], a solution of hydrotris(1H-pyrazol-1-yl)methane (0.47 mmol) in acetonitrile was added dropwise to a solution of dichloro(1,5-cyclooctadiene) palladium (0.47 mmol) in acetonitrile. The mixture was stirred at room temperature for 24 h, causing the solution’s color shift from yellow to light orange. The resultant precipitate was washed using diethyl ether and the obtained yield was 65%. Elemental analysis required for C_10_H_10_Cl_2_N_6_Pd: C 30.67, H 2.57, N 21.46; found: C 30.50, H 2.41, N 20.21%. FAR-IR (CsI pellet) (cm^−1^): 332 sp and 349 sp [ט (Pd-Cl)].

### 3.3. N-Formylation and N-Methylation of Amines with CO_2_ and NaBH_4_, Catalyzed by C-Scorpionate Metal Complexes

The reaction was conducted in a 10 mL glass pressure reactor. Sodium borohydride (1.5 mmol), amine (1 mmol), solvent (0.5–2 mL), and catalyst (1–5 mol%) were added to the reactor in the desired quantities. Once prepared, the reactor was pressurized with carbon dioxide to a pressure of 5 bar.

The reactor was then heated with constant stirring at either 30 or 80 °C for a desired reaction time. After reaching the reaction time, the reactor was cooled down, and the organic products were extracted using 15 mL of ethyl acetate (3 × 5 mL extractions).

The extracted products were subsequently analyzed using GC-FID (Gas Chromatography-Flame Ionization Detector) and GC-MS (Gas Chromatography-Mass Spectrometry), using a Shimadzu QP2010SE instrument (Column: Zebron ZB-5ms (30 m; 0.25 mm) Phenomenex). Substrate conversion and product yields were calculated from the surface areas of the corresponding signals, assuming that the sum of the areas 1a + 2a + 3a = 100%. Moreover, nuclear magnetic resonance (NMR) spectroscopy analysis was performed on a Bruker 500 MHz spectrometer, utilizing CD_3_CN (at 1.94 ppm) as the deuterated solvent.

## 4. Conclusions

The present work explored the catalytic performance of three C-scorpionate tris(1H-pyrazol-1-yl)methane type metal complexes in the N-formylation and N-methylation of amines with carbon dioxide and sodium borohydride.

It was observed that N,N-dimethylaniline and N-methyl-N-phenylformamide can be simultaneously obtained under mild reaction conditions, using N-methylaniline as substrate. The nickel(II) C-scorpionate complex, [NiCl_2_(tpm)]·3H_2_O, acted as catalyst, showing promising results, with higher conversions achieved at higher temperatures. However, prolonged reaction times led to a decrease in selectivity towards N-methyl-N-phenylformamide. Increasing the amount of catalyst enhanced selectivity towards N,N-dimethylaniline.

Also, the catalytic efficiency of [PdCl_2_(tpm)] and [CoCl_2_(tpm)]·3H_2_O was evaluated. In the case of [PdCl_2_(tpm)], a good conversion and selectivity was observed, while the cobalt(II) complex, [CoCl_2_(tpm)]·3H_2_O, exhibited a poor performance.

The present reaction was monitored by ^1^H nuclear magnetic resonance, with the idea being to better understand how the selectivity of the reaction occurs. This study also revealed the presence of an intermediate species that influenced the formation of the N,N-dimethylaniline. Interestingly, peaks correspondent to hydrogen, consistent with the use of sodium borohydride, and formic acid, promoted by the hydrogen formed in situ with the carbon dioxide, would contribute to the formation of N-methyl-N-phenylformamide.

A substrate scope was performed, in which aniline, piperidine, and diphenylamine were tested, using the optimal conditions achieved. The lowest conversion was obtained using diphenylamine as substrate, which could be explained by the presence of bulky phenyl groups in the molecule, when compared to the other studied substrates.

The catalytic system (NaBH_4_/MeCN/CO_2_) studied using homogeneous metal complexes proved to be effective for the functionalization of amines using carbon dioxide as C_1_ chemicals source.

## Figures and Tables

**Table 1:** N-formylation and N-methylation of N-methylaniline with CO_2_ and NaBH_4_, catalyzed by C-scorpionate metal complexes ^[a1]^.

Entry	Catalyst	Time (h)	Temperature (°C)	V_solvent_ (mL)	Conversion 1a (%) ^[c]^	Yield (%) ^[c]^		Ratio 2a/3a	TON	TOF (h^−1^)
						2a	3a			
1 ^[b]^	-	24	30	-	1.6	-	1.6	0/1	-	-
2 ^[c]^			30	0.5	29.4	16.8	12.6	0.57/0.43		
3			30		67.4	18.8	48.6	0.28/0.72		
4		8	30		54.7	21.4	33.3	0.39/0.61		
5		4	30		52.6	25.5	27.1	0.48/0.52		
6			80		91	36.9	54.1	0.41/0.59		
7	[CoCl_2_(tpm)]·3H_2_O	4	30	0.5	25.1	11.2	13.9	0.45/0.55	25.1	6.3
8	[NiCl_2_(tpm)]·3H_2_O		30		33.3	16	17.3	0.48/0.52	33.3	8.3
9	[PdCl_2_(tpm)]		30		32.2	14.7	17.5	0.46/0.54	32.2	8.1
10	[NiCl_2_(tpm)]·3H_2_O		80		85.6	27.1	58.5	0.32/0.68	85.6	21.4
11	tpm		80		88.9	37.2	51.7	0.42/0.58	88.9	22.2
12	[NiCl_2_(tpm)]·3H_2_O	24	30		65	21	44	0.32/0.68	65.0	2.7
13	[NiCl_2_(tpm)]·3H_2_O		80		97.6	45.6	52	0.47/0.53	97.6	4.1
14	[NiCl_2_(tpm)]·3H_2_O	4	80	2	81.6	23.6	58	0.29/0.71	81.6	20.4

^[a1]^ Conditions: 1.5 mmol NaBH_4_, 1 mmol N-methylaniline, 5 bar of CO_2_, 1 mol% of catalyst and acetonitrile. ^[b]^ 0.5 mmol of Et_3_N as additive and 1,4-dioxane; ^[c]^ 0.25 mmol of potassium carbonate (base) as additive. ^[c]^ Calculated by GC-MS.

**Table 2:** N-formylation and N-methylation of N-methylaniline with CO_2_ and NaBH_4_, catalyzed by C-scorpionate metal complexes ^[a2]^.

Entry	Catalyst	Time (h)	Temperature (°C)	V_solvent_ (mL)	Conversion 1a (%) ^[b]^	Yield (%) ^[b]^		Ratio 2a/3a	TON	TOF (h^−1^)
						2a	3a			
1	[NiCl_2_(tpm)]·3H_2_O	4	30	0.5	12.8	8.9	3.9	0.70/0.30	2.56	0.64
2	[NiCl_2_(tpm)]·3H_2_O		80		55.8	27.3	28.5	0.49/0.51	11.16	2.79
3	[NiCl_2_(tpm)]·3H_2_O		80	2	54.7	53.8	0.9	0.98/0.02	10.94	2.74
4	[PdCl_2_(tpm)]		80		78.7	23	55.7	0.29/0.71	15.74	3.94
5	[CoCl_2_(tpm)]·3H_2_O		80		33.4	18	15.4	0.54/0.46	6.68	1.67
6	[NiCl_2_(tpm)]·3H_2_O		30		24.9	17.3	7.6	0.69/0.31	4.98	1.25
7	[NiCl_2_(tpm)]·3H_2_O	24	30		37.1	10.1	27	0.27/0.73	7.42	0.31
8	[NiCl_2_(tpm)]·3H_2_O		80		48.7	18.6	30.1	0.38/0.62	9.74	0.41

^[a2]^ Conditions: 1.5 mmol NaBH_4_, 1 mmol N-methylaniline, 5 bar of CO_2_, 5 mol% of catalyst and acetonitrile. ^[b]^ Calculated by GC-MS.

**Table 3:** Substrate scope for N-formylation and N-methylation of amines with CO_2_ and NaBH_4_, catalyzed by C-scorpionate metal complexes ^[a]^.

Substrates	Products		
Aniline 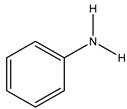	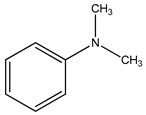 4%	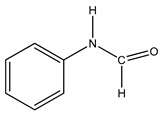 82%	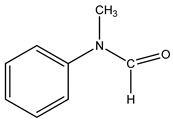 5%
Diphenylamine 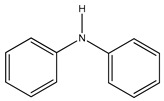	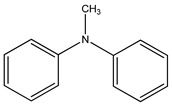 7%		
Piperidine 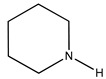	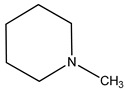 41%	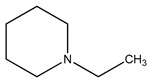 13%	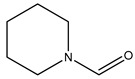 36%

^[a]^ Reaction conditions: 1.5 mmol NaBH_4_, 1 mmol substrate, 5 bar of CO_2_, 5 mmol% of [NiCl_2_(tpm)]·3H_2_O, 80 °C, for 4 h in 2 mL of acetonitrile. Calculated by GC-MS.

## Data Availability

The data presented in this study are available on request from the corresponding author.
